# Impact of temperature changes to the adhesion strength of molar tubes: an in vitro study

**DOI:** 10.1186/s12903-022-02144-y

**Published:** 2022-04-08

**Authors:** Benedikta Palesik, Kotryna Šileikytė, Julius Griškevičius, Rimantas Stonkus, Antanas Šidlauskas, Kristina Lopatienė

**Affiliations:** 1 Department of Orthodontics, Lithuanian University of Health Sciences, J. Lukšos - Daumanto str. 6, 50106 Kaunas, Lithuania; 2 Department of Biomechanical Engineering, VILNIUS TECH, J. Basanavicius Str. 28, Vilnius, Lithuania

**Keywords:** Orthodontic, Adhesive, Molar tubes, Temperature, Thermal cycle

## Abstract

**Background:**

The main purpose of this was to determine study adhesion strength of molar tubes bonding with a composite adhesive after exposure to a sudden change in temperature (thermal cycles).

**Methods:**

The study sample consisted of 40 recently extracted human first permanent molars, which were randomly divided into two groups of 20: group 1 was the experimental group (affected by thermal cycles), and group 2 was the control group. Molar tubes were bonded with a light-cure tube adhesive. The experimental group teeth were dipped 2,000 times in saline at 5 °C and at 55 °C. The control group were immersed in 37 °C saline. Molar tubes for both groups were removed with an adapted Mecmesim Multitesters 2.5—I, and the data were recorded with EMPEROR software. ANOVA was used to calculate and compare the results.

**Results:**

In the experimental group of the teeth, the maximum force was obtained at 94.2 N and the lowest force was 19.69 N. In the control group of the teeth, the maximum force was obtained at 159.1 N and the lowest force was 28.1 N. In the experimental group, the mean debonding force (59.12 N) was statically significantly smaller than in the control group (79.88 N), p = 0.0345. The forces in the control group were by 1.35 times greater than those in the experimental group.

**Conclusions:**

The forces of the adhesion of molar tubes to the tooth surface were reduced after exposure to a sudden change in temperature (thermal cycles). The results were significantly different between the experimental group and the control group.

## Background

Orthodontic treatment with fixed appliances improves facial aesthetics and oral function, which have a significant impact on both dental health and human psychological well-being [1]

During orthodontic treatment with fixed appliances, one of the main problems is bracket/molar tube detachment, as it can reduce the success of orthodontic procedures while increasing the duration and the cost of the treatment and damaging tooth enamel [2, 3] Studies of bracket/molar tube detachment have shown that the tubes detached from the molars more often than the brackets from the anterior teeth did [4–8]*.*

In order to optimise orthodontic treatment, various in vitro and in vivo studies have been performed to improve the quality of the bonding technique (direct and indirect) and to develop new adhesives based on the need to increase the shear bond strength, to shorten bonding time, to effectively reduce clinical bonding steps, and to preserve enamel. Orthodontic adhesives are divided into three major groups: chemical‐curing (glass ionomer cement, which sets by an acid‐base reaction), light‐curing (polyacid‐modified composite resin (compomer)), and a tri‐cure mechanism (resin‐modified glass ionomer cement) [5, 9–13]*.*

Studies have reported that clinical bonding should be considered to be successful when the minimum shear bond strength is 5.9–10 MPa, and the debonding of brackets/molar tubes should be performed easily and without damage to the enamel surface at the end of the treatment [14–16]. According to bonding protocols, the tooth surface is etched with 35–40% phosphoric acid before fixing the bracket/molar tube to ensure the desired dissolution of the surface enamel, which results in micro-porosity on the surface allowing the resin monomers to penetrate and mechanically bond. The bond strength obtained using this bracket/molar tube bonding protocol is generally high, ranging from 9 to 35 MPa [17]. Studies have shown that the bond strength in vivo was significantly lower than in vitro [18, 19].

Several factors affect the bond strength of orthodontic brackets/molar tubes, including contamination, type of the composite resin, viscosity of the adhesive, etching type of the enamel, storage conditions, size and shape of the bracket/molar tube base, temperature of the composite during bracket/molar tube fixation, enamel surface damage by caries or fluorosis, and restorations of the tooth [20]. Ingestion of food or beverages often causes sudden changes in temperature in the oral cavity, which also affects fixed orthodontic appliances, and thus it is necessary to study whether these temperature changes may affect the bond strength of brackets/molar tubes and to revise the orthodontic recommendations before treatment. To simulate the temperature changes in the oral cavity, thermally controlled water baths are used in in vitro studies [21].

However, a little research has been done on the effect of the thermal cycles on the adhesive after bracket/molar tube fixation and no studies have examined the correlation between bracket/molar tubes displacement and the force required during debonding. The formula of composites used in orthodontic for bonding brackets/molar tubes is improved every year. The aim of this in vitro study was to evaluate the effect of the thermal cycles on tube bonding with composite adhesion. Following this, a null hypothesis was formulated: there is no difference between the adhesives affected by thermal cycles and the control group in shear bond strength.

## Materials and methods

This in vitro study was performed in the Lithuanian University of Health Sciences at the Department of Orthodontic and the Laboratory of the Department of Biomechanical Engineering of VILNIUS TECH. The study was performed following an individual protocol, the methods of which were planned based on the previous studies [1, 22–26]. Bioethical approval was obtained from the University’s Bioethical Committee, No. BEC-OF-05.

Informed consent was obtained for experimentation with human teeth. The privacy rights of human subjects must always be observed.

The sample size of the trial was calculated according to the formula 1:

1 $${\text{n}} = \frac{{\left( {{\text{s}}_{1}^{2} + {\text{s}}_{2}^{2} } \right) \times \left( {{\text{z}}_{{1 - \frac{{\upalpha }}{2}}} + {\text{z}}_{{1 - {\upbeta }}} } \right)^{2} }}{{\Delta^{2} }}$$

n, the minimum sample size for each group; ${\text{z}}_{{1 - \frac{{\upalpha }}{2}}} = 1.96$ and ${\text{z}}_{{1 - {\upbeta }}} = 0.84$, when α = 0.05 and β = 0,2; ${\text{s}}_{1} ,{\text{s}}_{2}$, standard deviation of the first samples; and Δ, minimal clinically important difference.

Calculations were performed using G * Power (Version 3.1.9.2) statistical software. The following parameters were adjusted accordingly: significance level, 5%, strength test, 80%, standard deviation, 29.514 N and 20.755 N for pilot tests, and the least significant effect applied, 2.

e estimated minimum sample size required is 15 teeth for each group.

Tooth inclusion criteria were the following: molars with intact buccal enamel surface recently extracted for periodontal purposes, removed after jaw fractures, unsealed, not damaged by caries, not damaged by fluorosis, and without endodontic treatment. The teeth were collected over a month’s period. The extracted teeth were stored in a disinfectant (Gigasept Istru AF) for 15 min, then washed under running water for 1 min and kept in room-temperature (of 22 °C) saline according to the protocol of the previous tests in order to avoid a significant effect of the storage medium on the bond strength. The isotonic solution was changed daily to avoid bacterial growth.

After applying the selection criteria, 40 permanent teeth from a sample of 57 were included into our study. The teeth were randomly divided into two groups of 20, group 1 being the experimental group (E) (affected by thermal cycles), and group 2—the control group (C).

According to the protocol, the buccal surface of each tooth was polished for 30 s with non-fluoridated polishing paste and a rubber brush hand piece set at low speed, washed with water for 30 s, and blow-dried with compressed air for 10 s. the prepared enamel area was etched with 37% phosphoric acid for 40 s, washed with water for 30 s, and dried with compressed air until the tooth surface became non-glossy. Using a micro brush, the etched enamel surface was coated with a thin, even layer of binder resin (HIGH-Q-BOND BRACKET. PRIMER), and the air was blown until the binder became non-flowable. Tubes (American Orthodontic, ifit) were bonded to the centre of the clinical crown with a light-curing tube adhesive (HIGH-Q-BOND BRACKET), pressed with a 100 g weight on the buccal tooth surface and cured with a polymerisation lamp (Translux Wave, Heraeus Kulzer, Germany, 1000 mW/cm^2^) for 40 s, keeping the light source at 1 mm to the surface of the tube. The bonding was performed by one person to ensure accuracy.

Each tooth was centralised and fixed up to the neck area in iron rectangular boxes of the same shape filled with epoxy resin (Faserverbung) to ensure the stability of the samples. The molars were attached to the loom so that the buccal tooth surface would be parallel to the “Mechemsi” tension crushing device. All samples were numbered: the experimental group (affected by thermal cycles), E1-E20, and the control group of teeth, C1-C20.

Following that, 20 teeth of the experimental group were dipped 2,000 times in cold saline at 5 °C and hot saline at 55 °C. The immersion or dwell time in each bath was 30 s with a transfer time of 2–3 s. The saline temperature was maintained by a baby food heater/sterilizer. Meanwhile, 20 teeth of the control group were immersed in 37 °C saline. These prepared teeth were kept in saline at room temperature of 22 °C until the start of the test (i.e., for 5 h).

**Fig. 1:**
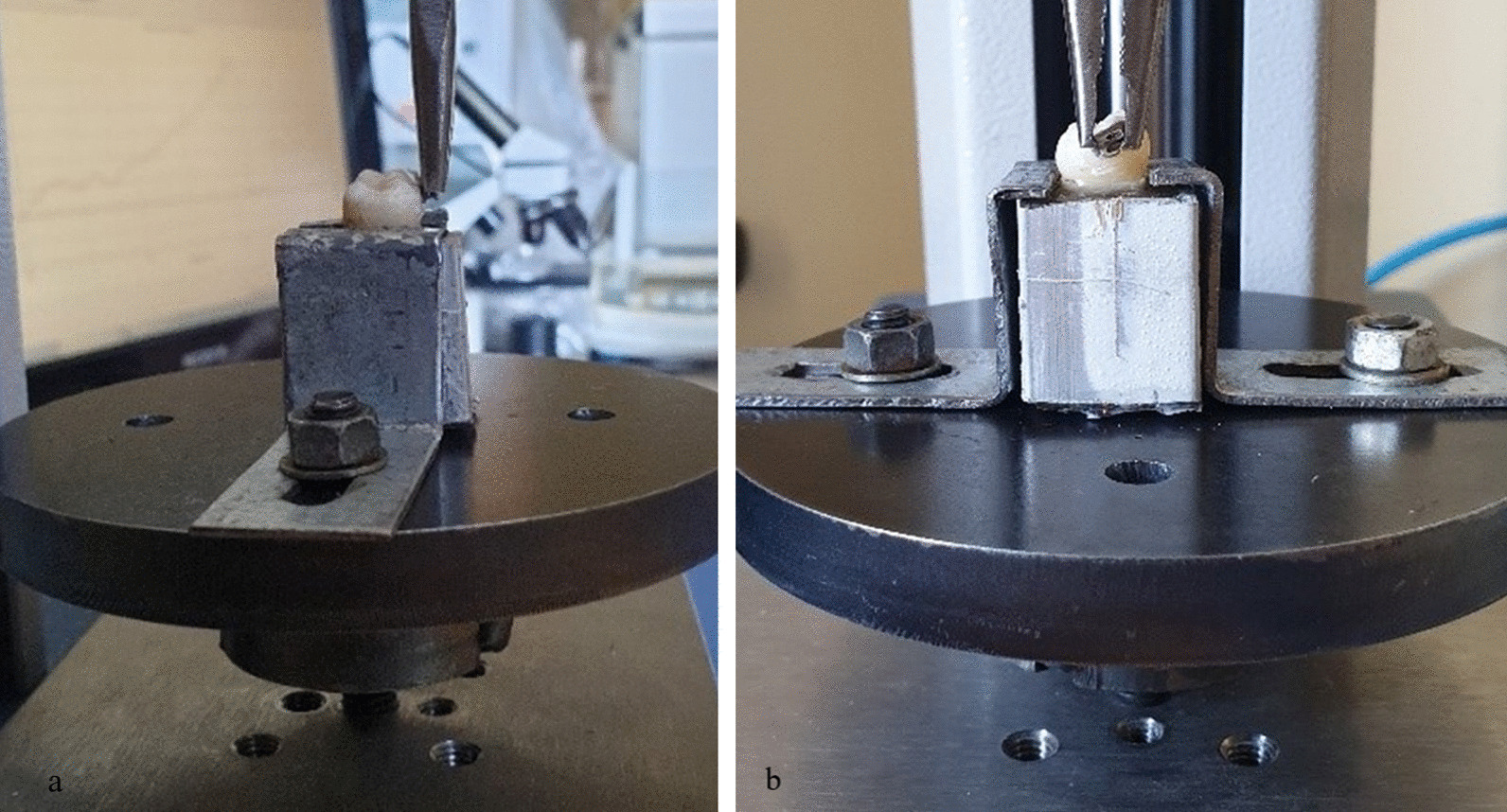
**a** Attaching teeth to the loom (Mecmesim Limited, United Kingdom) from the right side of the tooth. **b** Attaching teeth to the loom (Mecmesim Limited, United Kingdom) from the front of the tooth

The teeth were fixed on a loom. The pliers seen in the (Fig. 1) were customized to be fixed in an adapted Mecmesim Multitesters 2.5-I (Mecmesim Limited, United Kingdom) materials testing machine—welded screw to screw into the fixture and the pliers’ clamping was regulated with additional screw to ensure firm holding of each bracket. A tightening tube gripping mechanism and an epoxy-fixed tooth holder were fabricated for the attachment. The load applied was tensile: the bottom part of the tooth fixed in the epoxy was fixed to the bottom fixture plate and the upper part with the pliers was lifted. A load cell was fixed in between the pliers and the lifting mechanism of the materials testing device.

A constant speed of 0.1 mm/s was used to remove the tubes. This system was fully connected to the computer, thus the force and the displacement of debonding of the orthodontic tubes from the tooth surface was fixed. The adhesion of the molar tubes to the tooth surface was assessed by the debonding force and the displacement of the molar tubes during removal.

## Statistical analysis

The data were recorded with EMPEROR software. ANOVA (statistically used MATLAB program) was used to calculate and compare the results. One-way analysis of variance (ANOVA) was used to analyse the effects of temperature on the adherence of the tubes. The null hypothesis tested was whether the mean maximum debonding force of the tubes would be the same in the control and the experimental groups. Multiple comparisons using the Tukey–Kramer test were used to assess the significance of the mean difference. Statistical analysis was performed with the MATLAB (Mathworks Inc, USA) software package.

The difference in data between the control and the experimental groups was regarded as statistically significant when (p < α, α = 0.05).

## Results

**Fig. 2:**
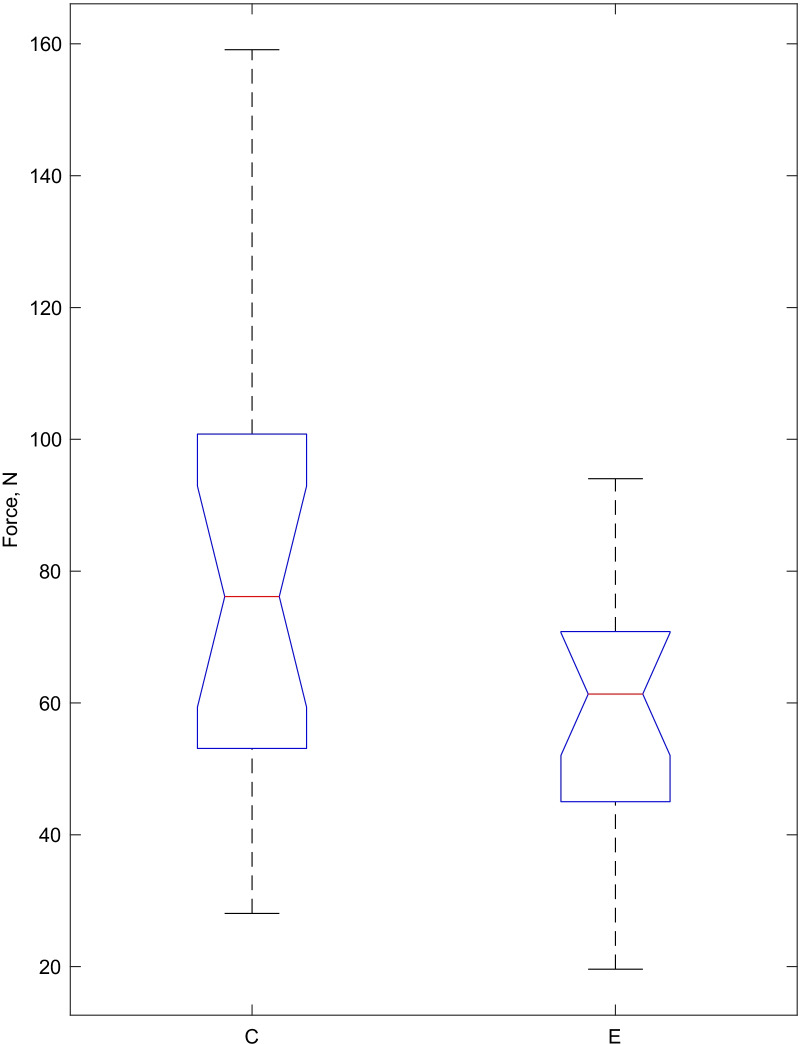
The results of mean, distribution, maximum, and minimum of debonding forces of E (the experimental group) and C (the control group). The mean of debonding force is indicated by red lines. The distribution of the debonding force is indicated by blue lines, and the maximum and minimum debonding forces are marked with black lines

In this study, we evaluated molar tube adhesion forces with tooth surfaces; The comparison of the debonding force and its distribution in the experimental and the control groups is presented in Fig. 2. In the experimental group, the mean debonding force (59.12 N) was statically significantly smaller than in the control group (79.88 N), (p = 0.0345), and thus the zero hypothesis was rejected.

**Fig. 3:**
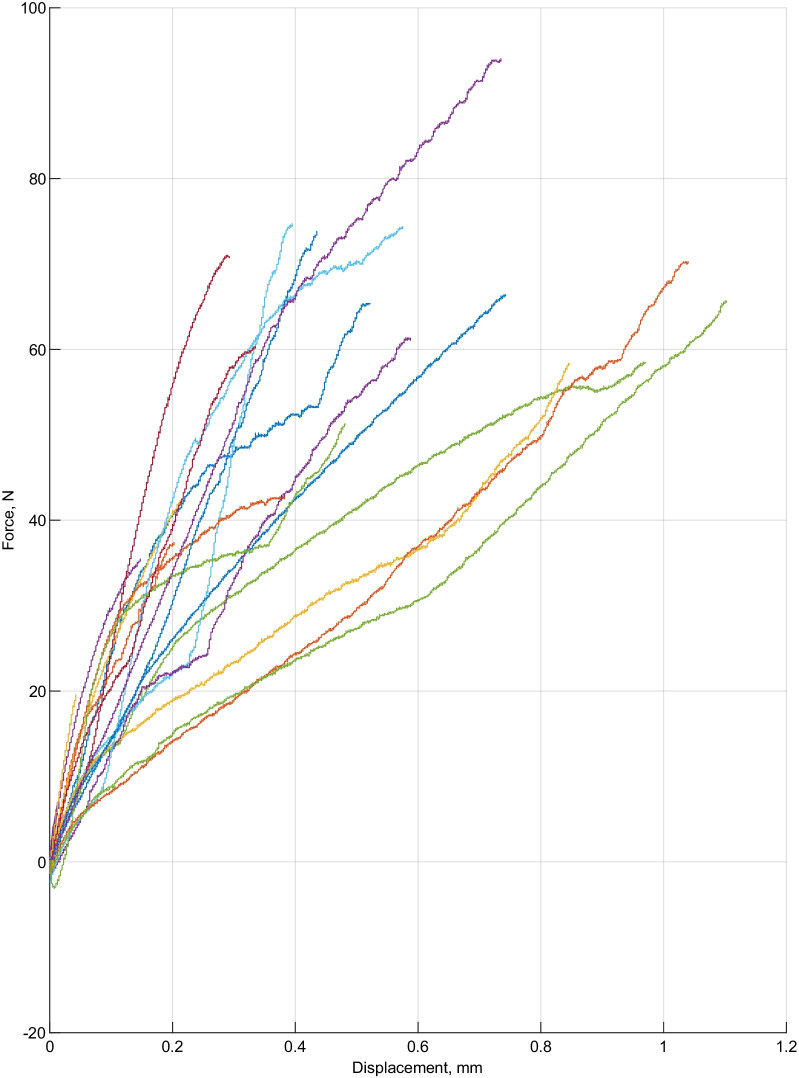
The dependencies of maximum debonding force and displacement in each tooth of the experimental group

**Fig. 4:**
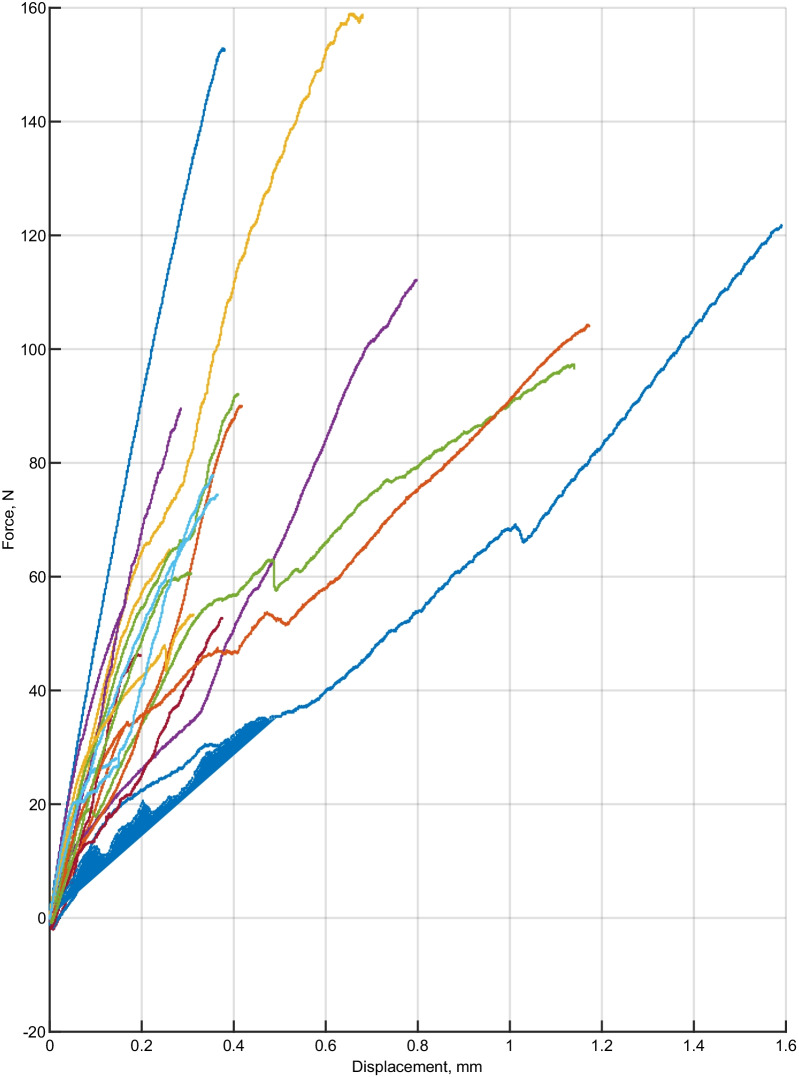
Dependencies of the maximum debonding force and displacement in each tooth of the control group

The results of force and displacement dependencies in the experimental are presented in Fig. 3 and the results of control groups are presented in Fig. 4. The purpose of this specific test is to determine the correlation between the displacement distance of the molar tubes and the force required during debonding.

In the experimental group of the teeth, the maximum force was obtained at 94.2 N with a displacement of 0,75 mm, and the lowest force was 19.69 N with a displacement of 0,08 mm. In the experimental group the maximum displacement (1,15 mm) was obtained at 62 N debonding force.

In the control group of the teeth, the maximum force was obtained at 159.1 N with a displacement of 0.69 mm, and the lowest force was 28.1 N with a displacement of 0.14 mm. In the control group the maximum displacement (1,6 mm) was obtained at 121 N debonding force. The mean displacement of the experimental group is 0,53 mm, the control group—0,47 mm.

## Discussion

To improve orthodontic treatment, evaluations of the factors that may impair bracket/molar tube adhesion are carried out. Laboratory tests are often used to evaluate the performance of adhesive systems before long-term clinical trials to determine the clinical efficacy of improved adhesive systems in the oral cavity [24]. The adhesion between the enamel and the bracket/molar tubes weakens due to the three different phenomena: mechanical, chemical, and thermal changes. Although in vitro studies cannot accurately reproduce in vivo conditions, when properly prepared and exposed, we can simulate different phenomena that occur in the oral cavity [30]. Thus, thermal cycling is fully accepted and widely used by the scientific community in experimental studies to create conditions similar to those in the oral cavity [12]. Even though some differences in temperature changes measured in the mouth and different tolerances for extreme temperatures have been reported, researchers agree that in laboratory tests on thermal cycle samples, the temperature should be from 5 °C to 55 °C [12]. These temperatures also comply with the technical specification of ISO TS 11,405 for testing the adhesion to the tooth structure [22]. The number of cycles in laboratory testing has not been based on scientific data [30].

The number of samples, the bracket/molar tube model, the time spent in the water baths, the transfer time, the number of cycles, and the removal techniques are chosen by the researchers, and the result of the adhesion of the brackets/molar tubes to the tooth surface can vary greatly depending on the method used. For this reason, Fritz with other authors proposed a separate control for each study [23].

It is important to mention that previous studies have shown that molar tubes debonded more often than brackets of premolars and anterior teeth (canines and incisors) [4–7]*.* In a year-long study of bracket/molar tube debonding frequency, Maijer and Smith showed that debonding rates of incisor, canine, premolar, and molar brackets/molar tubes were, respectively, 3.6%, 1.6%, 4.8%, and 11.6%, and it was reported that there was no significant difference of molar tube debonding rates between the first and the second molars [8]. For this reason, first molar teeth were used in this study.

In our study, while taking into account other studies [1, 22–26] the teeth of the experimental group were dipped 2000 times in 5 °C and 55 °C saline. The obtained results showed that the debonding force in the experimental group was, on average, by 20.75 N, or by 1.35 times lower than that in the control group. The statistically significant difference in forces of the composite (HIGH-Q-BOND BRACKET) between the control and the experimental groups was confirmed (p > alpha, p = 0.0345, alpha = 0.05).

In studies where the conventional bracket/molar tube bonding method was used [24], the mean values of bracket/molar tube adhesion to the tooth surface after 2000 and 5000 thermal cycles decreased slightly. These reductions were not statistically significant. However, Daub et al. [25] reported that the average adhesion of the brackets/molar tubes, using the conventional method of attaching the bracket/molar tube to human premolars and using Transbond XT adhesive, was significantly reduced by 16.7% after 500 thermal cycles. Saito et al. [26] observed a significant decrease in bracket/molar tube adhesion after 2000 and 5000 thermal cycles using the conventional bracket/molar tube attachment method.

Also in our study, using computer equipment, by debonding the molar tubes in the vertical direction, minimal displacement of the molar tubes until its complete detachment from the tooth surface was recorded. Such research has not been done in other studies. The displacement data of orthodontic brackets/molar tubes can be important in describing the viscously of the orthodontic adhesive system. Sho Goto et al. [27] shown that the orthodontic adhesives with high-viscosity and a high amount of filler have a higher bonding force of brackets. The same results are shown in the study of Andreas Faltermeier et al. [28].

There are no studies conducted to examine the correlation of orthodontic brackets/molar tubes resistance to displacement with debonding forces. Our study results show that the orthodontic molar tubes in the experimental group had a lower detachment force and a greater displacement during removal.

The control group of molar tubes had a higher detachment force and a smaller displacement during removal.

Based on the results of our and other studies [25, 26], an additional recommendation of orthodontist to the patient before orthodontic treatment should be included: to change dietary habits and not to cause sudden changes in the temperature in the oral cavity as this weakens the adhesion and increases the chance of debonding of the brackets/molar tubes.

Regarding the causes of the deterioration of the adhesion of the brackets/molar tubes, it is important to mention that during the thermal cycle test, the samples undergo sudden temperature changes and are exposed to water. Differences in the coefficient of the thermal expansion of metal brackets/molar tubes, the adhesive, and the tooth result in repetitive shrinkage/expansion stresses [12, 31]*.* The resin expands and contracts because its coefficient of thermal expansion is higher than that of the teeth; the higher the coefficient of thermal expansion of the resin, the worse it would be for the adhesive bond, as the volumetric changes in the resin will be greater. In addition, the water in this procedure causes hygroscopic expansion as well as chemical decomposition of the resinous components, which is called plastification [32, 33].

It has been suggested that the minimum bracket/molar tube adhesion force should be 6–10 MPa to achieve an acceptable clinical result [15]. From 1975, a standard procedure in the orthodontic practice is to etch the tooth surface with 35–40% phosphoric acid before bonding the brackets/molar tubes to ensure the micro-porosity on the tooth surface to allow the resin monomers to penetrate and mechanically bond and to increase the adhesion strength of the brackets/molar tubes from 9 to 35 MPa [1]. Our results far exceeded these limits. The difference could be due to the choice of different protocols and molar tube removal methods [17].

In vitro studies such as this have some limitations. Saliva and patients’ oral hygiene, diseases, and habits may affect the results, but in vitro, we cannot accurately simulate a multifactorial oral environment, thus the results of our study show the effect of only one factor on the debonding force of orthodontic brackets/molar tubes. In addition, some other factors, such as the type of the adhesive used, the mechanical and chemical surface preparations, and the number of thermal cycles may also affect the adhesion of the brackets/molar tubes. Thus, in order to obtain more accurate results and to evaluate and compare the effect of the thermal cycles on different types of adhesives, a greater number of subjects and studies are required. The effect of the thermal cycles on the adhesion strength of ceramic brackets/molar tubes to the tooth surface should also be evaluated. In addition, in order to continue research into the displacements of orthodontic braces during detachment, the fastening system should be improved to obtain accurate data.

## Conclusion

The adhesion force of molar tubes to the tooth surface can be significantly reduced by continuous thermal changes. For this reason, when discussing the recommendations of orthodontic treatment with fixed appliances for patients, the influence of temperature changes on the adhesion of the brackets should be discussed as well. Orthodontic patients with fixed appliances may be advised not to mix hot and very cold foods / drinks such as ice cream with coffee, iced water, and hot meals at the same time as eating/drinking, as such temperature changes reduce the adhesion force of braces / molar tubes to the tooth surface and increases of orthodontic bracket / molar tubes debonding.

## Data Availability

All data generated or analysed during this study are included in this published article.
